# Infant Damnation

**Published:** 1890-02

**Authors:** 


### INFANT DAMNATION.

The general assembly of the Presbyterian denomination of Christians having voted that the Creator has been long misrepresented in respect to His want of mercy in consigning to everlasting torment untutored heathen and helpless infants, has called for a modification of the creed in these particulars, but the Diocese of New York have been gravely considering the subject with no little opposition to any change of doctrine, and yet these learned bigots, domestic and imported alike, enjoin upon their hearers, the LOVE of God!

